# Germinal Rodríguez: public health, politics and medical popularization in the first person, Argentina, 1922-1960

**DOI:** 10.1590/S0104-59702024000100027

**Published:** 2024-06-17

**Authors:** Federico Rayez

**Affiliations:** i Becario Doctoral, Consejo Nacional de Investigaciones Científicas y Técnicas; Universidad Nacional de Quilmes. Bernal – Provincia de Buenos Aires – Argentina federicorayez@gmail.com

**Keywords:** Biography, Public health, Argentina, Medical popularization, Germinal Rodríguez (1898-1960, Biografía, Salud pública, Argentina, Divulgación sanitaria, Germinal Rodríguez (1898-1960

## Abstract

This article examines the career of Argentine doctor Germinal Rodríguez, situating it within the context of social history of medicine and the recent trend of medical biographies. Using a qualitative documentary analysis methodology, we analyzed various sources, including official records from the University of Buenos Aires, journalistic articles, and books by Rodríguez himself. Our analysis reveals that Rodríguez’s enjoyed a successful academic career in university teaching, while concurrently engaging in active socialist activism between 1920-1930. Beyond academia, Rodríguez served as a science popularizer, a policy consultant for his party, and even a public official during the Peronist era.

## Resumen

Este trabajo reconstruye la trayectoria del médico argentino Germinal Rodríguez en diálogo con la historia social de la salud y la enfermedad y con una reciente corriente historiográfica de biografías médicas. En base a una metodología cualitativa de análisis documental, analizamos expedientes oficiales de la Universidad de Buenos Aires, fuentes periodísticas, libros de Rodríguez y otras fuentes secundarias. Como resultado, podemos afirmar que su vida profesional estuvo marcada por la enseñanza universitaria y una exitosa carrera académica, así como por su intensa militancia socialista entre 1920-1930. Rodríguez fue también un divulgador, un experto de consulta en políticas públicas para su partido y funcionario estatal en los años del peronismo.

Germinal Rodríguez (1898-1960) fue un médico argentino, profesor de la Universidad de Buenos Aires, higienista especializado en asistencia social e impulsor del desarrollo de la carrera universitaria de servicio social en el país. Rodríguez desarrolló una intensa trayectoria como docente y también escritor, militante político, redactor de numerosas notas en la prensa partidaria del Partido Socialista Independiente (PSI) y en la prensa masiva. Los últimos años de su carrera incluyeron una intensa labor como asesor gubernamental durante los años del peronismo (1946-1955) en el área de medicina preventiva y social del Ministerio de Salud Pública. Su *performance* no estuvo exenta de polémicas y enfrentamientos políticos, tanto en el ámbito universitario como en la arena pública, donde acompañó a los socialistas independientes entre fines de los años 1920 y durante toda la década posterior como concejal en la Municipalidad de Buenos Aires. Se trata de una trayectoria que surcó las estructuras, instituciones y acontecimientos que se han estudiado la historia social de la salud y la enfermedad en América Latina (Armus, 2007; Cueto, Palmer, 2015; Carter, 2012; Ramacciotti, 2009); es decir, es una biografía relevante para la historia de la salud, de la configuración de las políticas sanitarias y de los progresos en materia de salud pública en Argentina.

La reconstrucción de su vida profesional es una tarea pendiente, apenas explorada por algunos trabajos (Sánchez, 2007, 2009; Becerra Solá, Becerra, 2009). Otros socialistas del mismo período, con claros intereses sociosanitarios, como Alfredo Palacios, Juan B. Justo y Enrique Dickman, han llamado la atención a los historiadores, mereciendo diferentes trabajos (García Costa, 1986; Souza, Hurtado, 2008). Incluso médicos contemporáneos a Germinal, destacados en los mismos ámbitos (los debates públicos de salud, la universidad, la función pública o la lucha política), tales como Juan Lazarte, Leopoldo Bard y Carlos Alvarado, también han sido indagados por la investigación histórica en los últimos años (Ledesma Prietto, 2016; Sánchez Antelo, 2012; Carter, 2012, p.107-140; Kohn Loncarica, Agüero, Sánchez, 1997).

La trayectoria de Rodríguez no ha recibido suficiente atención, y creemos que su biografía puede enriquecer una corriente de trabajos académicos que analizaron las vidas de médicos, científicos y sanitaristas desde la historia social de la salud y la enfermedad (Álvarez, Carbonetti, 2008; Nenadic, 2010; López González, Sáez Gómez, 2015; Buschini; Zabala, 2015; Ramacciotti, 2018; Batista, 2019; Campos, Carrijo, 2019; Serapioni, 2019; Hirschbein, 2020). Estos trabajos incrementaron nuestro conocimiento historiográfico de las personas que impulsaron políticas sanitarias, descubrimientos científicos o innovaciones institucionales, en la Argentina del siglo XX, aportaron información sobre nuevos hechos y aspectos en torno a procesos que fueron comprendidos desde un punto de vista estructural, como la formación de una dependencia estatal, la emergencia de una disciplina o especialidad, la profesionalización de las artes de curar etc. Las biografías médicas han puesto el acento en el carácter multifacético de las trayectorias, el hecho de abarcar en su desarrollo una variedad de campos sociales y estar atravesadas por diferentes momentos, lógicas institucionales y dinámicas políticas.

En el caso argentino, la indagación histórica desde la biografía individual y colectiva también ha mostrado la importancia metodológica del estudio de las carreras y las vidas como puertas de entrada al conocimiento de un campo intelectual y profesional (Balán, 1991; Ramacciotti, 2008; Galeano, Trotta, Spinelli, 2011; Buschini, Zabala, 2015; Ramacciotti, Rayez, 2019).

En función de esta perspectiva, que combina biografía e historia social creemos que una reconstrucción de la trayectoria del médico Germinal Rodríguez puede dar respuesta a algunos interrogantes. ¿Cómo podemos caracterizar esta trayectoria profesional? ¿Cuáles fueron los aportes concretos y la perspectiva desarrollada por este médico? ¿Cómo fueron sus experiencias como político, divulgador, higienista y de qué manera se conjugaron? Estas preguntas apuntan a recrear analíticamente las instancias más importantes de la vida de Rodríguez y aquellas que lo convierten en un caso interesante y a la vez muy ilustrativo del *milieu* social en el que vivió: su recorrido académico entre la higiene, la asistencia social y la medicina social; su agitado derrotero político entre el socialismo, primero, y el peronismo más tardíamente; la búsqueda de incidencia pública a través de la divulgación sanitaria. Como veremos, la combinación de estas actividades, actitudes políticas y comportamientos eran comunes en el campo médico, pero en el caso de Rodríguez mostraron una forma muy particular de anudarse.

Para arribar a algunas respuestas, utilizamos una metodología cualitativa de análisis documental que nos permitió rearmar, a partir de indicios y evidencias fragmentarias, la trayectoria de este personaje. Nos basamos en documentos oficiales de la Universidad de Buenos Aires (UBA), fuentes periodísticas (de la prensa masiva y la prensa socialista), libros escritos por Rodríguez, revistas especializadas en higiene y bibliografía especializada en la temática.

## Entre la Facultad de Ciencias Médicas y las disputas socialistas

### Sus primeros años

Germinal Rodríguez nació en 1898 (Sánchez, 2007, p.569). Fue uno de los cuatro hijos de Fructuoso Rodríguez Pardal, de ocupación comerciante en la ciudad de Buenos Aires (List of Manifest..., 1926; Kraft, 1942, p.66). Dos hermanos de Germinal fueron reconocidos médicos: Mercedes Libertad (nacida en 1902) y Oscar Rodríguez Rey (Sánchez, 2009). Asistió al Colegio Nacional del Sud (Bernardino Rivadavia), del cual egresó en 1915 (Legajo n.15.799..., s.f.). Un año después fue admitido en la carrera de ciencias médicas de la Universidad de Buenos Aires.

El ámbito médico porteño tuvo una larga etapa formativa durante el siglo XIX, desde la creación de la primera escuela universitaria en los años 1820 hasta la consolidación de la medicina diplomada como única arte de curar reconocida por el Estado, en los años 1870 y 1880 (González Leandri, 1999). Luego de la epidemia de fiebre amarilla que afectó a Buenos Aires en 1871, los saberes médicos fueron de vital importancia para la modernización y el saneamiento de la ciudad (Murillo, 2000). En los años 1870 se creó, en la Facultad de Medicina, la primera cátedra de higiene (Sánchez, 2007), la que, junto con el avance de la teoría bacteriológica de la enfermedad y los comienzos de la investigación de laboratorio, daría pie al nacimiento de una tradición higienista local, de corte positivista, ampliamente influyente en el diseño de políticas estatales en salud, en educación y en otros ámbitos.

Hacia las décadas de 1910 y 1920, cuando Rodríguez se formó como médico, la oferta académica en materia de higiene y medicina social apenas era diferente de la disponible unas décadas antes, pero suficiente para estimular rápidamente una vocación que el joven estudiante fue configurando. Según su legajo, obtuvo calificaciones altas en materias como “higiene médica” (9/10) y “bacteriología”, pero también en materias más tradicionalmente bio-médicas, como “clínica de enfermedades infecciosas”, “histología normal” y “clínica neurológica” (Legajo n.15.799..., s.f.). Hacia 1922, Rodríguez recibió su título de doctor en medicina luego de la presentación de su tesis, *Determinación de las condiciones higiénicas de la leche: medidas de higiene municipal* (Rodríguez, 1922). Esta preocupación sería recurrente en los años siguientes y sería la base de artículos y columnas como “Condiciones higiénicas de la leche” (en 1929), “El problema de la leche higiénica” (en 1930), “El abastecimiento de la leche en la ciudad de Buenos Aires” (en 1931) y “El problema de la leche” (Sánchez, 2007, p.569-571. Además de la salubridad de la leche como problema médico-social, Rodríguez mantuvo durante décadas una preocupación por la alimentación popular, opinando a través de diferentes medios sobre la importancia dietética de los productos lácteos en el consumo de la población (Rodríguez, 1935a, 16 abr. 1932).

Como ha señalado Buschini (2016, p.138), en los años 1920 y 1930, “diversas cuestiones asociadas a la producción, la comercialización y el consumo de alimentos se erigieron a la vez como objeto de indagación científica y como problema público”. Esto llevaría a la creación de un Instituto de Nutrición, en 1928, una Escuela Municipal de Dietistas, en 1933, una cátedra de “clínica de la nutrición” en la Universidad de Buenos Aires, en 1936, y finalmente un curso especializado de médico dietólogo en 1938, también en la UBA (Buschini, 2016). Sin embargo, el interés de Germinal por la alimentación no lo condujo a especializarse y convertirse en un referente de la temática, como los médicos Pedro Escudero o más tarde Boris Rothman. A diferencia de estos, Germinal fue sumando otros tópicos a su formación. En el tratamiento de otros temas como la invalidez y los accidentes laborales, Germinal Rodríguez dio muestras de estar conectado con otra variedad de temas relevantes para su época y sociedad. En este sentido, desde fines de la década de 1920 y durante toda la década de 1930 escribió numerosos trabajos sobre las cajas de seguro sociales, la asistencia social al minusválido, la discapacidad y las enfermedades laborales (Rodríguez, 1928, 1929, 1934, 1941). Estos temas fueron frecuentemente abordados por escrito, un hábito de comunicación de ideas que permitió a Rodríguez perfilarse como un higienista profesional y como divulgador sanitario.

Entre fines de la década de 1920 y principios de 1930, Rodríguez realizó varios viajes a Europa y Estados Unidos, para conocer instituciones de enseñanza de higiene y servicio social. En 1926, realizó un primer viaje a New York, junto a su esposa, Braulia Rodríguez (List of Manifest..., 1926), y, en 1930, emprendió una gira más amplia en la que visitó las Escuelas de Salud Pública de las universidades Johns Hopkins (Baltimore, EEUU), Yale y Boston. En este viaje también conoció el Servicio Social del Hospital General de Boston y la School of Social Workers de New York. En Europa, visitó la London School of Hygiene and Tropical Medicine, el Instituto de Higiene de la Universidad de Hamburgo y distintos hospitales y servicios sociales de Alemania y Austria (Legajo n.15.799..., s.f.). En 1931, visitó Alemania con una beca de la Kaiser Wilhelm Gesellschaft, según informó *La Prensa*, de Buenos Aires (Médico argentino…, 31 mayo 1931). En Berlín, dictó una conferencia en la Harnack Haus, sobre los progresos de la Argentina entre 1810 y 1930 (Conferencia del..., 9 jun. 1931). En esta gira de observación y estudios, Rodríguez tenía el objetivo de ampliar sus conocimientos sobre salud pública y medicina social.

Podemos encontrar ejemplos de viajes similares en los años 1920 y 1930 en las biografías de médicos contemporáneos a Germinal, como David Sevlever (Rayez, 2017), Carlos Alvarado (Carter, 2012, p.107-140) y Ramón Carrillo (Ramacciotti, 2008). Se trataba de un *cursus honorum* bastante establecido en la comunidad médica de Buenos Aires en las primeras décadas del siglo XX y de un camino que el joven Rodríguez debió sentir como obligatorio si deseaba ingresar en el círculo de los especialistas en higiene, espacio académico que había sido el hogar de recordadas figuras, como Eduardo Wilde (1844-1913), Guillermo Rawson (1821-1890), Emilio Coni (1855-1928). En estos años, mientras Rodríguez intentaba abrirse paso en el reducido campo de la higiene pública, médicos como Gregorio Aráoz Alfaro (1870-1955) y Alberto Zwanck (1884-1958) se hallaban en el centro de la escena. Aráoz Alfaro fue director del Departamento Nacional de Higiene en 1923-1926 y en 1930-1931, escribió sobre higiene infantil y salud materno-infantil, lucha antituberculosa y asistencia social (Sánchez, 2007, p.515-516).

Alberto Zwanck fue titular de la cátedra de higiene y medicina social en la UBA entre 1931 y 1946. Además, fue organizador de la Escuela del Servicio Social del Museo Social Argentino; sus temas de interés fueron los acuerdos sanitarios, la calidad de las aguas y los métodos de potabilización por cloro, los problemas derivados del hacinamiento habitacional, las enfermedades profesionales etc. (Sánchez, 2007, p.548-549). Bajo la supervisión de Zwanck, Rodríguez comenzó, en 1923, su “adscripción” a la cátedra de higiene y medicina social (Legajo n.15.799…., s.f.). Años después, en 1932, fue nombrado “segundo jefe de trabajos prácticos” de la cátedra de higiene y medicina social, y en 1934 alcanzaría el puesto de “profesor adjunto”.

## Actuación en el socialismo independiente

Este acercamiento y ampliación de sus preocupaciones tendientes a adoptar la medicina social como su principal perspectiva intelectual, además de ser una opción académica, alimentada por sus viajes al exterior y por su incorporación a la cátedra de higiene, tenía correlato con su acercamiento orgánico al socialismo. Como afirmó en un relato sobre su viaje a la Unión Soviética en 1931, nació dentro del movimiento socialista de la Argentina (Rodríguez, 1935b), en referencia a que su padre, Fructuoso, era un simpatizante del partido de Justo, Repetto y Dickman.

A principios de los años 1920, Rodríguez comenzó su carrera política como militante del Partido Socialista (PS), en ese momento comandado por Juan B. Justo (1865-1928). Eran los años de la presidencia de Marcelo T. de Alvear, representante del ala más “aristocrática” de la Unión Cívica Radical, crecientemente enfrentada al ala “yrigoyenista”, más popular y más ceñida a la conducción de Hipólito Yrigoyen. Hacia 1927, una fracción del PS se escindió con Antonio de Tomaso a la cabeza para formar el PSI (Sanguinetti, 1987), de tendencia liberal y explícitamente contraria a la conducción “autoritaria” del PS. Entre otros líderes, este partido estuvo formado por Roberto Giusti, Augusto Bunge, Héctor González Iramain, Federico Pinedo, José Rouco Oliva, quienes también crearían el periódico ¡*Libertad*! (Prislei, 2005, p.239; Pérez Branda, 2007). Este nuevo agrupamiento se formó como una reacción a la conducción oficial socialista, y lanzó una plataforma similar a la del PS, pero atenta a principios socialistas moderados, cercanos al liberalismo económico y crítico de los liberalismos europeos y americanos de “raíces conservadoras”. Este ideario, convertido en plataforma de propuestas electorales en 1928, se inspiró en las ideas de Henry George (laborista estadounidense), Henri de Man (socialista belga), Emile Vandervelde (socialista belga) y otros socialistas moderados o “reformistas” como Karl Kausty (socialista alemán) (Pérez Branda, 2007; Prislei, 2005). Si bien los socialistas independientes rechazaban formas de organización colectivistas y corporativistas (asimilando el comunismo soviético y el fascismo italiano-alemán), creían en formas moderadas de control público de la economía, de los servicios públicos, así como en la nacionalización del petróleo y la limitación de la participación local de capitales ingleses y norteamericanos. Aunque algunas de estas ideas fueron defendidas por el gobierno de Yrigoyen de 1916-1922 y 1928-1930, los independientes apoyaron el golpe de Estado al líder radical en septiembre de 1930, en alianza con otros actores golpistas, como el general del Ejército Agustín Pedro Justo (1876-1943) – el diario *Crítica* de Natalio Botana (Sanguinetti, 1987, p.131-198).

Si bien Germinal aparentemente no participó de la conspiración que derrocó a Yrigoyen, sabemos que Antonio de Tomaso, su “padrino” político, tuvo un papel destacado. Por otro lado, desde 1931 y hasta fines del mandato del presidente Agustín P. Justo, varios miembros del PSI formaron parte del gobierno, como Antonio de Tomaso (ministro de Agricultura en 1932-1933) y Federico Pinedo (ministro de Hacienda desde 1933). Este hecho y el apoyo del PSI a un gobierno conservador y fraudulento causarían descontento y críticas en las filas del PS y en las propias bases de los independientes (Prislei, 2005, p.240-245).

Ahora bien, la participación de Germinal Rodríguez en esta experiencia lo llevó en 1928 a ser concejal en el Concejo Deliberante de Buenos Aires, junto a Carlos Manacorda y Manuel González Maseda (Pérez Branda, 2011, p.13), cargo que conservó hasta 1930, momento en el que renuncia a su banca una vez producido el golpe de Estado, y que renovó entre 1931 y 1935. Entre ambas experiencias, en 1930, fue nombrado interventor médico en el Cuerpo Médico de los Ferrocarriles del Estado (Rodríguez, 1935b). Asimismo, no fue el único médico en el socialismo independiente, pues también participaron sus colegas José Ciancio y Domingo Urizaga (Pérez Branda, 2007). Paralelamente, Rodríguez tuvo una participación muy activa en la redacción del periódico *¡Libertad!*. Este diario partidario, del cual Germinal fue accionista, comenzó a imprimirse en 1927 en los talleres del diario *Crítica*, perteneciente a Natalio Botana, y se mantuvo en diferentes formatos durante toda la década de 1930.

Desde fines de los años 1920, Rodríguez repartió su atención entre esta responsabilidad política como concejal y el ejercicio de su profesión como asesor en la Caja de Jubilaciones Ferroviarias (creada por la ley 10.650), un vínculo con el mundo del trabajo y el movimiento obrero que perduró en el futuro. La actuación de Rodríguez como concejal lo llevó a presentar múltiples proyectos de reforma de la asistencia social en la capital federal. En ese sentido, participó en debates sobre la financiación del servicio de salud pública de la ciudad, la Asistencia Pública; aconsejó la creación de un hospital para convalecientes, en 1929; planteó en varias oportunidades la creación de cajas de seguros sociales para afrontar el problema de la invalidez (Rodríguez, 1934). En septiembre de 1935, en ocasión de la inauguración del servicio de asistencia social del Hospital Alvear de Buenos Aires, Rodríguez pronunció un discurso, luego reproducido por *¡Libertad!*. Afirmó entonces que la asistencia social y el servicio social eran conceptos científicos, actividades de base científica “sujetas a normas y principios por fuera de los cuales no puede salirse y se impone en su acción los mismos principios que la medicina en cuanto la semiología es reemplazada por una amplia encuesta social, para llegar al diagnóstico social y con ella después al tratamiento social” (El nuevo…, 25 sep. 1935). Decía también que la asistencia social estaba dominada en la ciudad por la “imprevisión”, el “acaso”, el “capricho de sus ejecutores”. El problema principal, según Rodríguez, era el desorden, la falta de planificación, y por ende el derroche de recursos. La asistencia pública debía empezar por atender los problemas de las madres y los niños recién nacidos, y el servicio que se inauguró ese día era una respuesta a ese problema y había sido una propuesta de Rodríguez. Lejos de manifestarse conforme, Rodríguez afirmaba también que, dado que no había un criterio científico, tampoco se sabían fijar criterios para la asignación de las partidas presupuestarias, y, por lo tanto, la administración carecía de información estadística para asignar los recursos donde eran más necesarios: “No sabemos cuántos pudientes parciales o totales se asisten en forma gratuita” (El nuevo…, 25 sep. 1935) – refiriéndose a los pacientes que pudiendo pagar por servicios médicos se atendían gratuitamente en hospitales de la ciudad. Para cerrar su discurso, Rodríguez hacía un llamamiento a crear una asistencia social racional, inteligente y “restitutiva”, que ayudara al necesitado a levantarse para que pueda valerse por sus propios medios.

Este enfoque de la cuestión discrepa con otras posturas del campo médico argentino del período. Juan Lazarte, por caso, médico anarquista de la provincia de Santa Fe, también estaba profundamente preocupado por la cuestión organizativa y financiera de los servicios médicos. A Lazarte no le preocupaba tanto el “derroche de recursos” sino el avance de una medicina capitalista y estatal sobre la práctica médica y un deterioro de la salud pública y la salud de los trabajadores. El patrón profesional de Lazarte también era distinto: lejos de la función pública o la política de partidos, Lazarte apostó por la organización sindical de los médicos (Abad de Santillán, Invaldi, Cappelletti, 1964; Ledesma Prietto, 2016). Fue dirigente en su pueblo de Santa Fe, pasó después a la Federación Médica provincial y en los años 1940 escaló a la Confederación Médica de la República Argentina. Como líder sindical y como prolífico intelectual con muchos libros publicados en Argentina, México y España, Lazarte se opuso a la estatización de la medicina y a la transformación del médico en un funcionario e incluso se opuso al peronismo y a la política llevada adelante por Ramón Carrillo. Como veremos más adelante, otros médicos argentinos tenían opiniones similares, pero adoptaron una actitud más benévola frente al peronismo.

Las posiciones de Germinal giraban en torno a una mejora de la asistencia pública y a una ampliación de las metas de la higiene pública, para lo cual se necesitaba un trabajo de coordinación entre la sociedad civil y el Estado, y también un esfuerzo de transparencia y racionalidad en el gasto. Es decir, mantenía un discurso con finalidades socialistas, pero con una clara orientación liberal.

## Germinal como escritor: nuevas ideas y nuevas controversias

Ahora bien, las opiniones de Rodríguez se alimentaban muchas veces de la medicina social, un entramado discursivo en plena formación en los años 1930 (Carter, 2023, p.94-121), pero también de los principios socialistas, en lo relativo a la ayuda a los trabajadores, y principios liberales, comunes a otros miembros del PSI y que el médico tomaba de Henri de Man, socialista belga (Prislei, 2005). Otros referentes que aparecían en sus discursos, escritos y columnas eran el ya citado Vandervelde, el alemán Kautsky y otros referentes de la Segunda Internacional. Durante los años 1930, Rodríguez desarrolló argumentos políticos para justificar el accionar de su partido, cada vez más empantanado en un contexto de fraude electoral y corrupción, pero también continuó en la búsqueda de una perspectiva teórica clara y pragmática. Esta indagación, que realizaba desde la cátedra universitaria y se expresaba en la publicación de diversos libros y folletos, lo condujo a la elaboración de una perspectiva propia llamada “demophylaxia”, una propuesta que unificaba el campo de la higiene y la medicina social e invitaba a una superación de las corrientes en boga, incluida la eugenesia.

Los puntos de vista de Germinal eran frecuentemente volcados en el periódico *¡Libertad!* o en *El Mundo, Crítica* o diarios zonales como *El Eco de Barracas*, donde el médico polemizaba sobre diversos temas políticos, pero también daba a conocer los principios de la asistencia social y la medicina social, en una tarea de divulgador sanitario. Entre 1934 y 1935, Rodríguez participó, a través de estos medios de comunicación, de varias polémicas que afectaron su imagen pública y lo colocaron en el centro de sonoras discusiones. Ya en 1930, *La Vanguardia*, el periódico oficial del PS, respondía a un folleto que Germinal había publicado bajo el título de “La crisis política del socialismo argentino: Partido socialista o partido de vanguardia”. En su prólogo, si bien criticaba al entonces presidente Yrigoyen, reconocía los aportes de los radicales a la democracia y a la política de masas; también elogiaba al presidente en la medida que se oponía a una aristocracia elitista, una “oligarquía”. La columna de *La Vanguardia*, en la que se tildaba a Rodríguez de “libertino de fuste” (mote que utilizaban para denostar a los socialistas independientes y su periódico *¡Libertad!*), acusaba al médico de colaborar con el yrigoyenismo, contradiciendo la plataforma de su partido y señalando sus incongruencias. En *Noticias Gráficas*, también solían criticar al concejal Rodríguez. En un número de febrero de 1932, una caricatura representaba al “cabeza de lista” de los socialistas independientes, el doctor Rodríguez, y se lo veía con una jeringa en la mano y vestido con un delantal blanco. El epígrafe remataba la imagen con la expresión: “Dr. Germinal Rodríguez, voluminoso *leader* de los socialistas independientes en el Concejo Deliberante” (Cabeza de…, 15 feb. 1932).

Sin embargo, en 1934, Rodríguez participó de otras polémicas que lo llevarían eventualmente a la ruptura con su partido. Su trabajo con mutualidades obreras y las cajas jubilatorias (como la de los ferroviarios), y la preocupación por la falta de solvencia financiera de las mismas, quedaron plasmados en *La invalidez: como un problema médico-social y su profilaxis por medio del seguro social*, de 1934, un libro en el que acusó públicamente al médico higienista Augusto Bunge (1877-1943), diputado por el PSI y hasta hacía poco compañero de ideas, por la “bancarrota” de la caja de jubilaciones de los obreros ferroviarios. Esto sucedió un año después de la muerte de Antonio de Tomaso, líder de los independientes y factor de unidad de personalidades muy diversas. Como era de público conocimiento la contabilidad de las mutualidades era un tópico preocupante y objeto de malestar en el sistema de salud desde la década de 1920. En ese entonces, Bunge preparó un informe en el que se detallaban las fallas de recaudación, la sobre carga de servicios y consecuentemente la insolvencia (Belmartino, 2005, p.72-80). Los dichos de Rodríguez ocasionaron una enérgica respuesta de Bunge, quien atacó al médico tanto en la universidad y como en público a través de la prensa partidaria. Como primera medida envió al decano de la Facultad de Ciencias Médicas una encendida nota en la que resumidamente acusaba a Rodríguez de mentir sobre la responsabilidad del diputado en la crisis de las cajas jubilatorias y mutuales. De *La invalidez...* decía que constituía una “calumnia” y un “desacato”, un “fárrago de transcripciones y comentarios improvisados, y tergiversaciones que no me detendré en detallar” (Legajo n.15.799..., s.f.). Para Bunge, los ataques de Rodríguez solo eran una triquiñuela destinada a conseguir un mejor sueldo como empleado de la Caja de Jubilaciones ferroviaria, un buen pretexto para crear una sección especial dentro de la institución.

Para finalizar, Bunge le solicitaba al decano (doctor Rafael A. Bullrich) que informara si la obra era una publicación oficial del Instituto de Higiene (por lo tanto, sustentada con fondos públicos), y pedía explicaciones sobre el hecho de que el libro llevaba el membrete oficial de la facultad y de la cátedra de higiene. También interrogaba al decano si las opiniones de Rodríguez representaban el pensamiento del titular de la cátedra, Alberto Zwanck, del mismo decano y del consejo directivo de la facultad. Según consta en el mismo expediente oficial de la Facultad de Medicina, la respuesta de Zwanck fue que el trabajo había corrido por cuenta del propio doctor Rodríguez, toda vez que el Instituto no disponía de fondos para costear publicaciones. Además, en esencia, el libro *La invalidez* sostenía ideas compartidas por la cátedra (como la “Profilaxis por el Tratamiento”), y por ese motivo se publicaba con el membrete oficial. Como resultado, la Comisión de Interpretación y Reglamento dictaminó hacia fines de 1934 que en adelante las obras de contenido “contencioso” y “polémico” no podrían ostentar marcas, sellos ni membretes oficiales de la Facultad (Legajo n.15.799..., s.f.).

El ataque de Augusto Bunge continuó en el periódico *¡Libertad!*. En agosto del mismo año, el camarada de Rodríguez envió al diario socialista independiente una carta abierta dirigida a Héctor González Iramain, su compañero de bancada parlamentaria y presidente de la Caja de Jubilaciones de los ferroviarios (Una carta…, 26 ago. 1934). En el escrito, igualmente violento que la carta enviada al decano de medicina, Bunge llamaba a Rodríguez “un empleado de la Caja”, y afirmaba que luego de casi veinte años de conocer la problemática de las cajas y mutualidades había logrado presentar un proyecto de reforma para sanear sus finanzas; para Rodríguez, según este *racconto* de Bunge, este proyecto iba a descapitalizar las cajas. Bunge respondía entonces que su proyecto llegaría a lograr un superávit de 22 millones de pesos para las cajas y que Rodríguez o no había entendido la propuesta o solo le interesaba inventar mentiras para lograr escalar en la caja ferroviaria, una maniobra que lo hacía quedar como un héroe. La carta finalizaba con amenazas de acciones penales contra Rodríguez.

**Figura 1:**
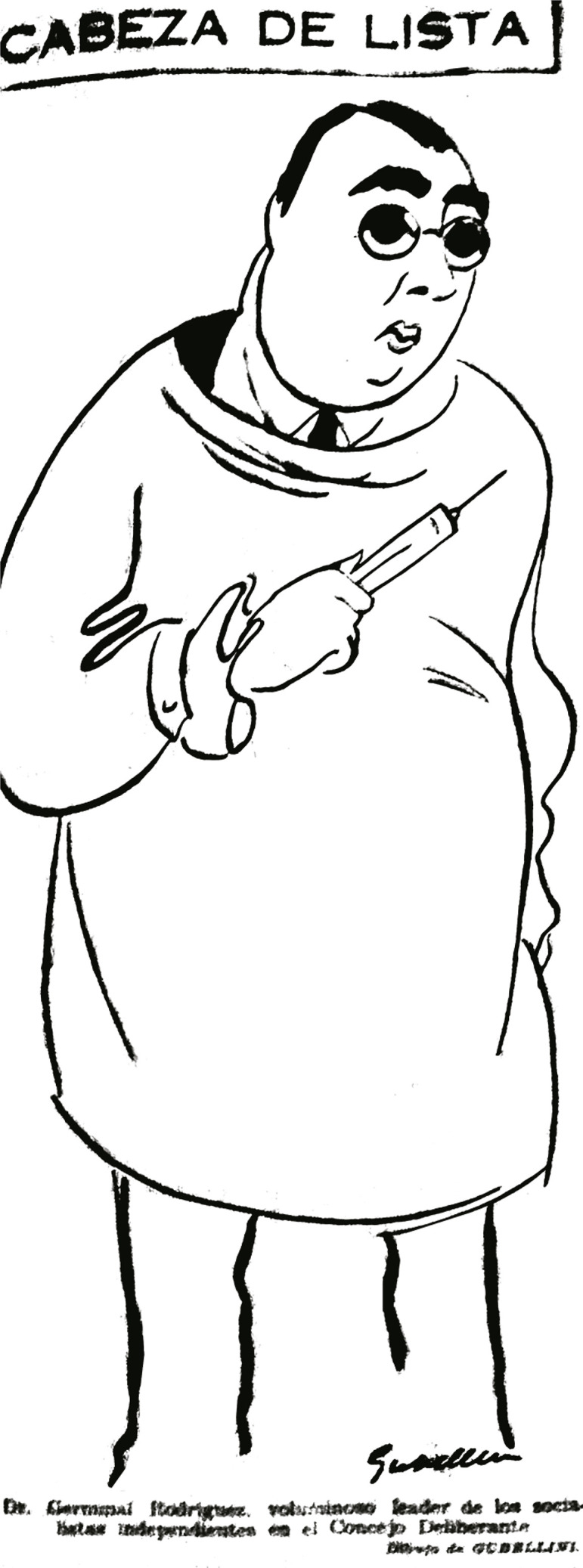
: Dr. Germinal Rodríguez, voluminoso leader de los socialistas independientes en el Concejo Deliberante (*Noticias Gráficas*, 15 feb. 1932)

Unos días después, la respuesta de Rodríguez se publicó en el mismo diario. En una nueva carta, decía que su libro era claro y que no necesitaba reproducir sus argumentos. Acusaba a Bunge de “inconducta política” impropia de un diputado nacional (El dr. Germinal…, 28 ago. 1934). Y argumentaba que el espíritu de su propuesta coincidía con el proyecto del diputado Padilla, quien proponía incorporar a la ley de la Caja Civil “el derecho [del trabajador] a reintegrarse al trabajo cuando el estado de invalidez desaparece, situación legal olvidada por el especialista Bunge, y que toleró durante 20 años de parlamentario semejante exacción a esa caja en bancarrota” (El dr. Germinal…, 28 ago. 1934). Este es un punto interesante de la polémica porque toca una fibra profunda del pensamiento de Rodríguez: la búsqueda de equilibrio y racionalidad en los gastos de la previsión y la asistencia social. Como afirmó Prislei (2005), Rodríguez era un seguidor de Henri de Man, socialista belga, autor entre otras obras de *Plan du travail* y consecuentemente planteaba un alejamiento del marxismo puro y una opción por el “solidarismo”, un socialismo de apoyo mutuo, basado en los sindicatos (“socialismo de guildas”). Este punto no resulta tan claro consultando otras obras, donde Germinal se muestra favorable a cualquier tipo de colectivismo, incluido uno basado en el poder estatal (Rodríguez, 1935b, p.27), lo cual nos habla de ciertos matices, contradicciones o pragmatismo ideológico quizás presentes en su manera de pensar.

También resulta interesante la polémica con Bunge porque nos habla de disputas encarnizadas por la legitimidad del saber acerca de lo social, es decir, luchas por quién puede hablar sobre los problemas sociales y sanitarios, quién detenta la autoridad para analizar, nominar y legislar sobre el fenómeno, quién puede emitir juicios intelectuales competentes acerca de dicha materia etc.

En este punto, hacia 1935, las ideas y planteos de Rodríguez se habían distanciado cada vez más de la dinámica de su partido. En una sucesión de notas compiladas en *Sociocracia y socialismo independiente* (Rodríguez, 1935b), como “Política a la deriva”, “Hacia un nuevo socialismo”, “A los afiliados del PSI” etc., Rodríguez se distanció gradualmente de la dirigencia del partido, llamó a una redefinición de la doctrina y la táctica del PSI y acabó realizando un balance negativo de la deriva partidaria en los últimos años: estancamiento, “alianzas con trepadores”, olvido de los principios partidarios y del programa, apoyo a un gobierno que actuaba “contra el salario de los trabajadores” etc. (Prislei, 2005, p.244). En los años siguientes, Rodríguez y otros militantes se alejaron definitivamente del socialismo independiente pero no de los problemas sanitarios y sociales que los convocaban intelectual y académicamente.

## Profesionalización de la higiene y acercamiento al peronismo

Hacia principios de los años 1940, la Facultad de Ciencias Médicas de la UBA abrió varias especializaciones nuevas, como el título de “médico higienista” (Sánchez, 2007), psiquiatría (Balán, 1991), medicina forense, tisiología, infectología, anestesista (Buschini, 2020). Este escenario de cambios en el mundo universitario dio a la higiene y la medicina social y preventiva un marco muy propicio para acentuar un proceso de profesionalización. En 1938, durante el sexto Congreso Nacional de Medicina, Alberto Zwanck, titular de la cátedra de higiene, y Alfredo Sordelli, bioquímico y bacteriólogo, presentaron un cuadro de la formación en higiene en la universidad (Zwanck, Sordelli, 1938). Al finalizar su ponencia insistieron en la necesidad de una escuela o instituto para la formación de higienistas. En este sentido, la creación del “curso superior de higiene” en 1941 articuló una demanda que había comenzado a crecer en las décadas anteriores.

La carrera de Germinal Rodríguez estuvo estrechamente vinculada a esta innovación institucional. Hacia estos años era profesor universitario en Buenos Aires, pero también en el Colegio Nacional de Buenos Aires había sido titular de la materia higiene pública y profesor de higiene en la Universidad Nacional de La Plata (Legajo n.15.799..., s.f.). Asimismo, 1941 coincidió con su ascenso como “jefe de trabajos prácticos” en la cátedra de higiene. Según su legajo, tuvo que esperar otros seis años para ocupar el puesto de profesor titular. La creación del curso superior significó para Rodríguez un nuevo lugar desde el cual ejercer la enseñanza de la higiene, la medicina social y el servicio social. A su vez, los egresados del curso pronto formaron una Asociación Argentina de Higiene, con su propia publicación periódica, la revista *Hygieia* (Biernat, Ramacciotti, Rayez, 2019). La Asociación y su revista fueron canales de articulación muy importantes entre el ámbito de la higiene y la salud pública y el nuevo gobierno que se instaló en 1946. La gestión peronista impulsó una serie compleja y densa de políticas sanitarias que hicieron necesaria la presencia dinamizadora de expertos y cuadros técnicos formados en higiene y administración de la salud (Ramacciotti, 2009). La Asociación de Higiene proveyó muchos cuadros al nuevo gobierno, entre los que se encontraban varios profesores de la cátedra de higiene de la UBA, como Luis Lepera, Homero Rodríguez Cámpora, el propio Germinal Rodríguez y otros cuadros que pronto ocuparon las líneas técnicas de la Secretaría de Salud de la nación comandada por el doctor Ramón Carrillo. El pasaje del doctor Rodríguez del socialismo independiente de los años 1930 al peronismo desde 1946 es un episodio que permanece inexplicado, y no hemos hallado indicios en las fuentes sobre tal giro, pero parece razonable a la luz de la conformación “amplia” del peronismo como movimiento político-ideológico en sus años tempranos, momento en el cual se incorporaron al naciente Partido Justicialista ex-radicales, conservadores, nacionalistas y ex-socialistas. El propio Enrique Dickman, dirigente histórico del PS, quien permaneció en el partido incluso después de la ruptura de los Independientes, se acercó al peronismo en 1952 encabezando un efímero Partido Socialista de la Revolución Nacional.

Asimismo, el arribo de Rodríguez a la Secretaría de Salud Pública puede haberse tratado de una incorporación tanto político-ideológica como técnica: un puesto de poder permitiría llevar a la práctica una serie de reformas e ideas que rondaban los discursos médicos desde varias décadas atrás y que se asentaban en una gran insatisfacción de estos agentes frente a la insuficiencia del sistema sanitario y a su incapacidad para afrontar las demandas de atención médica. Los artículos y panfletos escritos por Rodríguez en los años 1930 apuntaban en general contra una política social y sanitaria irregular, inarticulada y muy por debajo de las necesidades populares. Lo cierto es que el médico higienista ocupó el cargo de secretario general de medicina preventiva, en 1946, posición que fue cambiando de denominación a lo largo de los años pero siempre en la misma área temática.

Leopoldo Bard también se incorporó al gobierno peronista. Al igual que Germinal, había estudiado medicina en la UBA, ocupó diversos roles en diferentes cátedras y se involucró en la militancia de la Unión Cívica Radical desde el primer gobierno de Yrigoyen en 1916. Actuó como diputado durante los gobiernos radicales, entre 1916 y 1930, proponiendo numerosos proyectos de ley, incluido el primer proyecto de tratamiento sobre consumo de drogas (Bard, 1957; Sánchez Antelo, 2012). También como Germinal publicó muchos trabajos de divulgación sanitaria, siendo pionero en la historia de la medicina en el Río de la Plata. Entre 1947 y 1955 trabajó como asesor de la Secretaría de Trabajo y Previsión (Ministerio de Trabajo), creando reglamentos y códigos para regular la atención a la salud de los trabajadores en el ámbito sanitario.

Como decíamos más arriba, no todos los médicos interesados en la salud pública como problema público adhirieron o se plegaron al proyecto peronista. Juan Lazarte se opuso a la política sanitaria del peronismo y fue crítico de lo que llamaba el peligro de burocratización. Pese a esto, espacios como la Asociación Argentina de Higiene (AAH) nuclearon a una gran diversidad de médicos y médicas de higiene pública del país, facilitando intercambios que, en el ámbito público, e incluso en la universidad controlada por el peronismo y el nacionalismo católico, no se podían efectuar. En 1948, la AAH organizó el primer Congreso Nacional de Higiene y Medicina Social, al cual asistieron representantes de diferentes provincias, universidades, dependencias y centros de formación. La mesa de medicina del trabajo, por ejemplo, contó con la participación de Leopoldo Bard, Juan Kaplan, de la cátedra de higiene de la UBA y Mercedes Rodríguez de Ginocchio, por el Museo Social Argentino y también hermana de Germinal. La sección de seguridad social contó con la presencia de Juan Lazarte, ya mencionado, David Sevlever, médico de Santa Fe, y Oscar Rodríguez Rey, por el Museo Social Argentino, hermano de Germinal. Muchos expertos que tuvieron luego carreras muy exitosas y largas participaron de este encuentro, como Francisco Menchaca, Noel Sbarra, Alberto Yanzón, así como figuras de renombre como Carlos Alvarado, Gregorio Aráoz Alfaro y Alberto Zwanck. Germinal participó de este congreso representando a la cátedra de higiene (ya como titular) y como afiliado a la AAH.

Con la llegada del peronismo al poder en 1946, el ministro Carrillo llevó adelante una política sanitaria basada en la idea de “unidad de comando” de un sistema sanitario complejo. Según Ramacciotti (2009), desde la Secretaría de Salud Pública, creada en 1946, se intentó lograr la sistematización administrativa de los servicios sanitarios, coordinar los organismos dispersos, compensar el desequilibrio entre Buenos Aires y las provincias y modernizar la burocracia encargada de gestionar en materia sanitaria. Estas metas habían sido buscadas desde fines del siglo XIX por los funcionarios del Departamento Nacional de Higiene; desde 1943 se reiteró el intento centralizador desde la Dirección Nacional de Salud Pública (Biernat, 2015). El ministro Carrillo llevó adelante una política sanitaria ambiciosa centrada en la mejora de la atención hospitalaria, mediante la construcción de un nuevo entramado hospitalar, centros de salud, clínicas y sanatorios (generales, especializados, para niños, ancianos etc.). Otros elementos importantes de la gestión fueron las campañas sanitarias para combatir epidemias y las misiones sanitarias nacionales que organizaron la distribución de insumos médicos y la concreción de estudios de población; las políticas de control de la salud del trabajador en ámbitos fabriles y rurales; la educación sanitaria en las escuelas, centrada en la promoción de una alimentación saludable.

Estas iniciativas fueron coordinadas desde el ministerio, y a través de sus 34 direcciones generales, en su mayoría coordinadas por médicos. Estas estuvieron abocadas a diferentes áreas de intervención sanitaria, por ejemplo, direcciones de medicina asistencial, de industria farmacéutica y farmacia, de hospitales generales, de hospitales psiquiátricos, de medicina sanitaria, de enfermedades tropicales, de tuberculosis, de higiene pública etc. (Ramacciotti, 2009, p.85). Germinal Rodríguez ocupó la Dirección de Medicina Preventiva y el Consejo de Medicina Preventiva. Su intervención fue eminentemente intelectual, aportando ideas y lineamientos que podemos ver reflejados en sus artículos de los *Archivos de la Secretaría de Salud Pública* (Rodríguez, 1946, 1947, 1948a, 1949). Por ejemplo, en 1948, Rodríguez aprobaba la creación de la Escuela Superior Técnica en Salud Pública; según sus palabras, un acontecimiento que “sale del marco común de los hechos diarios” (Rodríguez, 1948a, p.127-138). Instaba a redoblar esfuerzos para superar las limitaciones del saber higiénico. Según su visión, la higiene, tal como se practicaba hasta la década de 1940, padecía de varios defectos, “quedaba resumida en la ingeniería sanitaria y en la epidemiología”, a las que consideraba superadas. La primera porque era una disciplina autónoma, y la segunda “porque ya llenó su objeto” (p.133). En tanto disciplina científica, quedaba encerrada en los claustros universitarios y era vulnerable de “academismo”: “El higienista puro, de libro, pierde contacto con la realidad social y, su ciencia se vuelve tan extraña al ambiente en que actúa que impresiona a los ojos de sus colegas como un ser venido de otro planeta” (Rodríguez, 1948a, p.135). Por otro lado, en aquellas experiencias la higiene “se reducía a la simple aplicación del poder de policía” en contraste con lo que debería ser una moderna medicina social, basada en la solidaridad y en los métodos preventivos (p.131).

**Figura 2:**
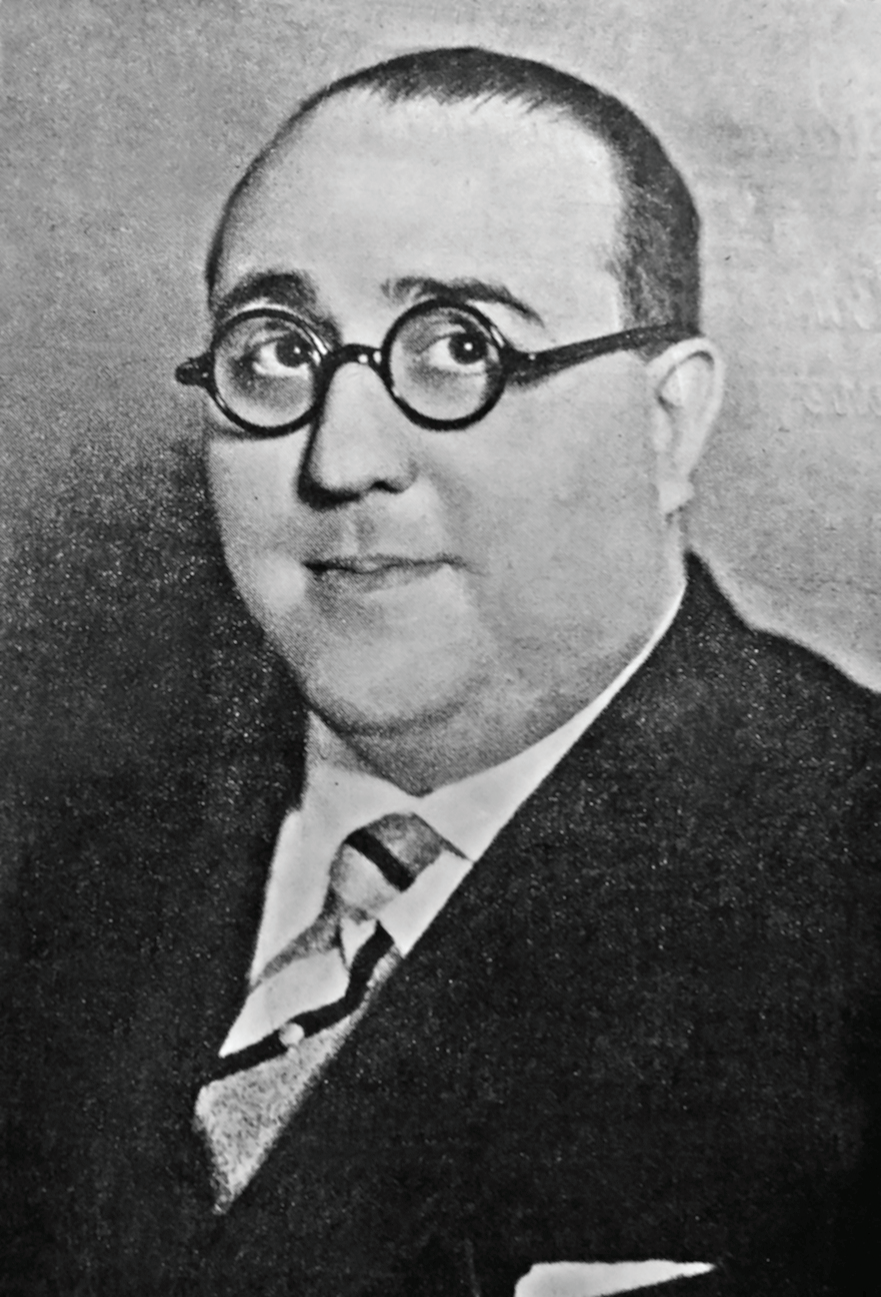
: Fotografía de Germinal Rodríguez (Professor…, 1947)

El aporte conceptual de Rodríguez fue importante durante todo este período, como asesor del gobierno, pero también como profesor y divulgador: dictó cursos libres parciales de higiene y medicina social (1944), de medicina del trabajo (1947), de educación sanitaria (1947, junto al ministro de trabajo José M. Freire y el médico Leopoldo Bard) (Legajo n.15.799..., s.f.). Como comunicador, aunque ya no contaba con la tribuna partidaria de *¡Libertad!*, continuó trabajando y publicando por otros medios. A través de editoriales populares Rodríguez dio a conocer más de cuarenta libros, entre otros, sus compendios de “demofilaxia”, o “demophylaxia”, tratados de higiene y servicio social. En Americalee, una editorial porteña dedicada especialmente a imprimir y difundir obras de divulgación, el médico publicó *Higiene pública* (1945), *Higiene individual y medio externo* (1945) (Sánchez, 2007) y *Compendio de demofilaxia* (Rodríguez, 1948b). Americalee, Editorial Claridad, Lautaro fueron algunos de los núcleos para la difusión del pensamiento médico‐social y la exhibición ante un público lego de los avances de la medicina en los años centrales del siglo XX. Así lo atestigua la participación de Juan Lazarte, otro médico importante en la época, en diferentes emprendimientos editoriales. Al igual que Germinal publicó en Americalee (*Problemas de medicina social*, 1943), pero también dirigió la Colección Eros de Editorial Partenón (1945-1963) y uno de sus libros más importantes, *La revolución sexual de nuestro tiempo*, en Editorial Nervio (1932).

Las obras de Rodríguez, como las de otros médicos que editaban sus obras por los mismos canales, trataban de explicar didácticamente en qué consistía la filosofía de una medicina social y preventiva, retomando aportes de teorías muy diversas desarrolladas en el campo de la medicina diplomada, la moderna epidemiología o la eugenesia. En *Compendio de demofilaxia*, Rodríguez afirmaba que hasta ese momento la higiene se había preocupado por el hombre enfermo para evitar la diseminación de su mal, sea por medios directos (higiene pública) o indirectos (higiene social); la salud y el enfermo eran la primordial preocupación de la higiene. La medicina social desplazaba el blanco de su mira del hombre enfermo, al sano y se preocupa de estudiarlo en su medio social (Rodríguez, 1948b, p.11). Los objetos de análisis de esta nueva perspectiva eran la “orientación y la selección profesional; la re-educación profesional de los lisiados (mutilados, ciegos, sordomudos); el régimen de los subsidios sociales y las cajas de compensación, la mutualidad, el ahorro, el cooperativismo, la educación cívica, el deporte” (p.11). Esta medicina social y preventiva, esta “demofilaxia”, abarcaba la curación, la prevención, el tratamiento etc. Esta noción envolvente y desbordante, que iba de la medicina del hombre enfermo al estudio del hombre sano se cimentaba en cuatro principios muy vinculados, por otra parte, con la política sanitaria del gobierno peronista: “1º el examen periódico; 2º la profilaxis por el tratamiento; 3º la educación sanitaria; 4º la readaptación social” (p.11). Estos principios debían dar forma al accionar de los médicos, que ya no serían “tratantes”, sino “profilantes”.

El fuerte acento en la prevención señala un punto de contacto con el discurso eugenésico, imperante en la época. Hasta donde nos es posible afirmar en base a la obra escrita de Germinal, ésto no fue un representante del enfoque eugénico y ni siquiera una figura menor dentro de ese movimiento. La eugenesia como perspectiva teórico-ideológica estaba presente en la Argentina desde fines del siglo XIX, y fue promovida y aceptada por médicos como Antonio Vidal, Víctor Delfino, Gregorio Aráoz Alfaro (presidente de la Sociedad Eugénica Argentina), pero también por figuras como Alfredo Palacios, Juan A. Senillosa etc. (Vallejo, ago. 2018). Como afirmó Armus (2016, p.151), se trató de un constructo ideológico adoptado por “políticos liberales y conservadores, socialistas, radicales y anarquistas, nacionalistas, fascistas y filo-nazi, médicos y psiquiatras, abogados y criminólogos, escritores y ensayistas”. El tema central de la eugenesia era el “mejoramiento de la raza” y el combate contra los “venenos raciales” que se transmitían por herencia, debilitando a las poblaciones. Aunque esta perspectiva tuvo expresiones más “genetistas” o más centradas en el ambiente, sus preocupaciones giraron en torno

los problemas sanitarios de la ciudad, la selección del inmigrante, la lucha contra los ‘venenos raciales’ – en primer lugar la sífilis, la tuberculosis … – la protección de la maternidad y la infancia, la creación de una conciencia eugénica mediante el autodisciplinamiento individual, la caída de la natalidad entre la población blanca y la declinación de la inmigración europea luego de 1930, es aislamiento y la segregación sexual de los irrecuperables … como modo de evitar el contagio y la procreación no deseada (Armus, 2016, p.151).

Esta ideología fue sostenida por la Sociedad Eugénica Argentina y luego por la Sociedad Argentina de Biotipología, Eugenesia y Medicina Social, formada en 1930, y desde estos espacios influyó en los ámbitos universitarios, especialmente en la Facultad de Ciencias Médicas de la UBA y la cátedra de higiene. La participación de Germinal en estas redes se dio distintas maneras. Por ejemplo, a través de su capital social más próximo: sus hermanos Oscar Rodríguez Rey y Mercedes Libertad Rodríguez de Ginocchio eran exponentes locales de la eugenesia; el primero como experto en higiene mental (Dovio, 2017), partícipe de núcleos eugénicos importantes como la Sociedad de Biotipología; y la segunda como figura de referencia en debates sobre reproducción, natalidad y maternidad (Ledesma Prietto, 2016, p. 26-31). Figuras ligadas a la cátedra de higiene de la UBA, en la que Germinal hizo una extensa carrera universitaria eran partidarios públicos de la eugenesia, como Gregorio Aráoz Alfaro. En la producción teórica de Rodríguez, si bien se destaca la construcción de un marco conceptual no estrictamente eugénico (centrado en la prevención, la profilaxis, la administración sanitaria, la “demofilaxia”), hay que mencionar también que pronunció discursos publicados por la revista *Anales de la Sociedad Argentina de Biotipología, Eugenesia y Medicina Social* (Rodríguez, 1940, p.13), y participó, junto a su hermano Oscar, del primer Congreso de Sociología y Medicina del Trabajo, organizado por esta Sociedad (Miranda, 2009). En 1927 también había publicado un artículo, “Registro de sanidad de la República”, donde expresaba “la pretensión de fichar ‘la vida y la salud de los habitantes, tal cual hoy se registra la vida y haciendas del pueblo’” (Miranda, s.f.). Asimismo, a pesar de este acercamiento a la eugenesia, no puede afirmarse que Rodríguez haya sido un eugenista. Más bien se trató de un higienista universitario que desarrolló la mayoría de sus ideas en el marco de su cátedra y al amparo que la higiene como disciplina le ofrecía.

A diferencia de otros médicos muy activos en el período y ya mencionados más arriba, como Juan Lazarte o Leopoldo Bard, Germinal desarrolló una larga carrera universitaria de manera ininterrumpida que culminó en lo más alto del poder universitario. Durante el período peronista conquistó la titularidad de la cátedra de higiene, en 1947. Alberto Zwanck se había retirado en 1946, y el primer puesto en el concurso fue para Rodríguez (Legajo n.15.799..., s.f.). Los médicos Teodoro Tonina y Carlos Carreño quedaron en segundo y tercer lugar respectivamente. Este patrón universitario exitoso no es común a otras trayectorias. Juan Lazarte halló la docencia universitaria hacia el final de su carrera, al terminar la experiencia peronista, cuando fue convocado para la normalización (Abad de Santillán, Invaldi, Capelletti, 1964). Por su parte, Leopoldo Bard vio interrumpida su carrera en 1930, cuando se produjo el golpe de Estado (Bard, 1957). Muchos otros médicos e investigadores, como Bernardo Houssay y Florencio Escardó, no pudieron continuar con sus carreras universitarias sino hasta la caída del peronismo. La titularidad de Rodríguez duró hasta la intervención de la universidad en 1955, con el golpe de Estado de septiembre de ese año. A partir de ese momento, la cátedra pasó a estar dirigida por Guido Ruiz Moreno, y la carrera de Rodríguez continuó en sus últimos años en otros medios.

## Últimos años de carrera durante el post-peronismo

La intervención de la universidad en 1955 abrió un proceso de recuperación de la autonomía universitaria y del gobierno tripartito compartido por profesores, estudiantes y egresados, y paralelamente estimuló la “desperonización” de las diferentes facultades (Buchbinder, 2005; Salomón, 2015). Mientras los cargos titulares fueron concursados, muchos profesores políticamente identificados con el gobierno peronista renunciaron o fueron despedidos. Otros tantos, que habían sido profesores hasta 1943-1946 y habían sido dejados cesantes o por la dictadura de 1943 o por el gobierno peronista en 1946, regresaron a sus facultades, como profesores titulares o como nuevas autoridades. En la Facultad de Ciencias Médicas de la UBA retornaron varios profesores: Florencio Escardó, que ocuparía el cargo de decano, acompañado de un grupo de colegas con un amplio reconocimiento en la universidad, Bernardo Houssay, Eduardo Braun Menéndez, Alfredo Lanari, Mario Brea, Guido Ruiz Moreno y Eduardo de Robertis. En este contexto de cambios, Germinal Rodríguez abandonó la Facultad de Medicina en 1955-1956, y el médico epidemiólogo Guido Ruiz Moreno ganó la titularidad de la cátedra.

En los años siguientes, últimos de su carrera, Rodríguez concentró sus actividades como profesor de la Escuela de Servicio Social del Museo Social Argentino. Esta Escuela se había creado en 1930 y desde entonces estuvo a cargo de Alberto Zwanck, el titular de higiene de la UBA y mentor de Germinal Rodríguez. Entre el plantel docente se contaba al propio Alberto Zwanck, a cargo de higiene social y economía política, en tanto Rodríguez estaba a cargo de demografía y estadística. Junto con biología humana, eran las materias del primero año del curso. Durante el segundo año los alumnos debían cursar y aprobar “técnica del servicio social”, “elementos de legislación social” y “patología social” (Sánchez, 2007, p.51‐53). Los primeros “trabajadores sociales”, como serían llamados luego, egresaron de esta Escuela en 1932 (p.50). Se trataba de una formación novedosa que intentaba formalizar el entrenamiento profesional de los asistentes sociales, por lo que Germinal fue reconocido años después como uno de los principales impulsores del servicio social profesional en la Argentina (Maiola, 2018). Según afirmaba el propio Rodríguez, se debía alcanzar mediante esta formación un entrenamiento integral, que capacitara a los egresados de esta Escuela ir más allá de la miseria y comprender las causas sociales y políticas de la pobreza. En una obra de 1948, decía por ejemplo que el servicio social comprendía “las medidas destinadas al desarrollo del bienestar del pueblo, desde los puntos de vista higiénico, económico, moral y educativo”, lo cual se pondría de manifiesta en acciones “destinadas no solo a los enfermos o necesitados sino a toda la masa de la población” (Rodríguez, 1948b, p.96-97). El servicio social podía definirse como “toda obra humana destinada al bien de los semejantes, con el propósito del bien mismo, sin esperar de ella usufructo, beneficio u honor, aun cuando su realización pueda reportar los mismos” (p.97). Estas palabras aparecían en su *Tratado de demofilaxia*, tanto en su edición de 1948 como en la de 1955, y dan cuenta de un giro en su manera de pensar la asistencia social: la misma era pensada como un servicio que no esperaba “usufructo, beneficio u honor” una noción bastante lejana a aquella sostenida por nuestro personaje en los años 1930, preocupado en ese entonces por la racionalidad de los gastos, el abordaje ordenado y científico de la asistencia pública etc.

Lo cierto es que la trayectoria política de Germinal Rodríguez, su carrera académica y su rol como divulgador y formador se concentraron en los últimos años, luego de su retiro de medicina en 1955, en la Facultad de Servicio Social del Museo Social Argentino, donde fue nombrado decano. Ocupó este cargo hasta 1960, cuando falleció a los 62 años. Su temprana muerte le puso final a una carrera muy prolífica en términos académicos, disciplinares, pero también como actor político y como divulgador público de los saberes médico-sociales que cultivó a lo largo de 40 años de trayectoria.

## Consideraciones finales

Planteamos aquí una exploración de la vida de un médico argentino más allá del consultorio. Como hemos visto, la trayectoria de Germinal Rodríguez constituyó un cúmulo de experiencias sociales, culturales y políticas muy amplias. Su vida dejó huellas en la higiene pública como disciplina académica, en la universidad, en la política socialista y la administración peronista, así como en la historia del servicio social y la divulgación sanitaria. La reconstrucción de su polifacética vida de médico consagrado a la medicina social nos ha permitido echar una mirada a procesos sociales más amplios, explorados por la historia social de la salud y la enfermedad en Argentina, y a reconocer patrones comunes a otras carreras médicas. Como muchos otros, siguió un *cursus honorum* académico en una facultad prestigiosa ingresando como adscripto a la cátedra de higiene en 1923; conoció los ámbitos locales de la higiene y la salud pública, pero también los internacionales, a través de viajes de formación y observación realizados entre 1926 y 1932. Publicó sus ideas en libros, revistas, diarios, panfletos para dar a conocer a públicos legos sus propuestas y perspectivas. Estas apuestas intelectuales fueron llevadas por nuestro personaje a la arena política en los años 1920 y 1930 a través del PSI. En ese lapso de su carrera pudimos rescatar algunas polémicas en las que se vio envuelto y algunas características de su pensamiento social y político. Vimos que nuestro personaje sostenía las ideas compartidas con otros socialistas independientes, principios igualitarios moderados, libertades individuales, economía capitalista controlada o supervisada por el Estado etc. Siguiendo la huella de sus opiniones nos encontramos con una serie de polémicas: Rodríguez se enfrentó a otros socialistas, como Augusto Bunge, fue criticado por socialistas de *La Vanguardia* y otros medios, y hacia el final de la experiencia del PSI se enfrentó a otros dirigentes pidiendo una refundación del partido. Esta veta política de Rodríguez casi es desconocida y constituye un dato importante y una característica compartida con otros médicos especializados en higiene pública o medicina social. Rodríguez adhirió con acciones, con su pluma y con sus ideas al gobierno peronista, al que probablemente veía como una oportunidad de resarcimiento para llevar a la realidad todos los proyectos de mejora social y sanitaria que habían sido propuestos durante décadas por los socialistas de diversas tendencias desde la acción parlamentaria. Entre 1946 y 1955, su carrera alcanzó la cresta de la ola: fue su punto más álgido y también determinó sus últimos años de carrera.

Considerando el período 1920-1960, podemos preguntarnos si esta trayectoria que acabamos de analizar constituyó un fenómeno típico del campo médico argentino o de un hecho anómalo y extraordinario. Como acabamos de ver, la vida de Rodríguez estuvo atravesada por acontecimientos (desde congresos académicos hasta golpes de Estado), instituciones (cátedras, sociedades, institutos), prácticas (la escritura, las actitudes públicas, las polémicas, la participación política) y discursos (como la medicina social, la higiene, la eugenesia) que afectaron las vidas de múltiples médicos en la época, de una manera u otra. En este sentido se trata de un itinerario en el que se combinaron elementos comunes a muchos otros médicos: los viajes formativos al exterior, el camino hacia el rol de catedrático, la divulgación médico-sanitaria, la participación política. Quizás uno de los principales elementos de diferenciación en el caso de Germinal haya sido su adhesión al peronismo entre 1945 y 1955, la cual es más llamativa dado su procedencia socialista y cuasi-liberal. Rodríguez, así como muchos otros médicos y médicas, prestó su apoyo al gobierno de Perón y a la gestión de Ramón Carrillo, mientras otros, como Lazarte y el grupo de la Confederación Médica de la República Argentina se opusieron enérgicamente. Un elemento muy particular de Rodríguez, y que lo distinguió frente a otros médicos y médicas, es que combinó una sólida carrera académica, una carrera política también extensa y una *performance* muy prolífica como divulgador y comunicador.

Ahora bien, la figura y las obras de Germinal Rodríguez fueron ignoradas luego de su muerte. Ni su obra escrita ni su trayectoria fueron reconstruidas más allá de las menciones en revistas y publicaciones especializadas en historia de la medicina. Sin embargo, sus ideas y su preocupación por el servicio social conservaron algún lugar en los trabajos de algunos de sus discípulos, como Francisco Martone, un médico que continuó orbitando en la formación de visitadores sociales en diferentes ámbitos en las décadas posteriores (Ramacciotti, Rayez, 2019). La memoria de Germinal Rodríguez perduró también en las historias del trabajo social en la Argentina y en el proceso de profesionalización que atravesó esta ocupación desde los años 1930 en adelante, un hito por el que se reconoce la colaboración activa de Germinal Rodríguez.
